# Supplementary material for: MetaQTL: a package of new computational methods for the meta-analysis of QTL mapping experiments
Source: BMC Bioinformatics. 2007 Feb 8;8:49. doi: 10.1186/1471-2105-8-49 (PMC1808479; doi:10.1186/1471-2105-8-49)
Supplement: Additional File 7 — MetaQTL Package : jar file and tutorial. This Zip archive contains both the MetaQTL JAR file and the files of the tutorial. [file 1471-2105-8-49-S7.zip › org.inra.metaqtl/doc/overview-tree.html]

Class Hierarchy


|  |  |  |
| --- | --- | --- |
| **Overview** \| Package \| Class \| Use \| **Tree** \| **Deprecated** \| **Index** \| **Help** | |  |
| PREV   NEXT | **FRAMES**    **NO FRAMES**     **All Classes** |  |


---


## Hierarchy For All Packages

**Package Hierarchies:**: org.metaqtl, org.metaqtl.adapter, org.metaqtl.algo, org.metaqtl.bio, org.metaqtl.bio.entity, org.metaqtl.bio.entity.adapter, org.metaqtl.bio.entity.bean, org.metaqtl.bio.entity.factory, org.metaqtl.bio.util, org.metaqtl.factory, org.metaqtl.graph, org.metaqtl.main, org.metaqtl.numrec, org.metaqtl.util

---

## Class Hierarchy

- java.lang.Object
  - java.util.AbstractCollection<E> (implements java.util.Collection<E>)
    - java.util.AbstractList<E> (implements java.util.List<E>)
      - java.util.ArrayList<E> (implements java.lang.Cloneable, java.util.List<E>, java.util.RandomAccess, java.io.Serializable)
        - org.metaqtl.bio.entity.**BioEntityCollection** (implements java.util.Collection<E>, org.metaqtl.bio.IBioEntity)
    - org.metaqtl.graph.**AnchoredElement**
      - org.metaqtl.graph.**Layer**
        - org.metaqtl.graph.**ChromLayer**- org.metaqtl.graph.**QtlLayer**- org.metaqtl.graph.**QtlPartitionLayer**- org.metaqtl.graph.**QtlTreeLayer**
      - org.metaqtl.bio.util.**BioCrossTypes**- org.metaqtl.bio.entity.**BioEntity** (implements org.metaqtl.bio.IBioEntity)
          - org.metaqtl.bio.entity.**BioEntityContainer** (implements org.metaqtl.bio.IBioEntity)
            - org.metaqtl.bio.entity.**AlleleContainer** (implements org.metaqtl.bio.IBioLocus)
              - org.metaqtl.bio.entity.**Locus** (implements org.metaqtl.bio.IBioLocus)
                - org.metaqtl.bio.entity.**Marker** (implements org.metaqtl.bio.IBioLocus)- org.metaqtl.bio.entity.**QTL** (implements org.metaqtl.bio.IBioLocus)
              - org.metaqtl.bio.entity.**GroupContainer** (implements org.metaqtl.bio.IBioGenome)
                - org.metaqtl.bio.entity.**GeneticMap**
                - org.metaqtl.bio.entity.**Individual**- org.metaqtl.bio.entity.**IndividualContainer** (implements org.metaqtl.bio.IBioPopulation)
                    - org.metaqtl.bio.entity.**Population** (implements org.metaqtl.bio.IBioPopulation)
                      - org.metaqtl.bio.entity.**CrossPopulation** (implements org.metaqtl.bio.IBioCross)
                    - org.metaqtl.bio.entity.**LocusContainer** (implements org.metaqtl.bio.IBioLGroup)
                      - org.metaqtl.bio.entity.**LGroup** (implements org.metaqtl.bio.IBioLGroup)
            - org.metaqtl.bio.entity.**Ontology** (implements org.metaqtl.bio.IBioOntology)- org.metaqtl.bio.entity.**OntologyTerm** (implements org.metaqtl.bio.IBioOntologyTerm)
          - org.metaqtl.bio.entity.adapter.**BioEntityAdapter** (implements org.metaqtl.bio.IBioAdapter)
            - org.metaqtl.bio.entity.adapter.**BioEntityCollectionAdapter** (implements org.metaqtl.bio.IBioAdapter)- org.metaqtl.bio.entity.adapter.**GeneticMapBeanAdapter** (implements org.metaqtl.bio.IBioAdapter)- org.metaqtl.bio.entity.adapter.**LGroupBeanAdapter** (implements org.metaqtl.bio.IBioAdapter)- org.metaqtl.bio.entity.adapter.**LocusBeanAdapter**- org.metaqtl.bio.entity.adapter.**OntologyBeanAdapter**- org.metaqtl.bio.entity.adapter.**OntologyTermBeanAdapter**
            - org.metaqtl.bio.entity.bean.**BioEntityBean**
              - org.metaqtl.bio.entity.bean.**BioEntityCollectionBean**- org.metaqtl.bio.entity.bean.**GeneticMapBean**- org.metaqtl.bio.entity.bean.**LGroupBean**- org.metaqtl.bio.entity.bean.**LocusBean**- org.metaqtl.bio.entity.bean.**OntologyBean**- org.metaqtl.bio.entity.bean.**OntologyTermBean**
              - org.metaqtl.bio.entity.factory.**BioEntityFactory**
                - org.metaqtl.bio.entity.factory.**MapChartFactory**- org.metaqtl.bio.entity.factory.**MapMakerFactory**- org.metaqtl.bio.entity.factory.**MapRawFactory**- org.metaqtl.bio.entity.factory.**MapTabFactory**- org.metaqtl.bio.entity.factory.**OntologyTabFactory**- org.metaqtl.bio.entity.factory.**XmlBioEntityFactory**
                            - org.metaqtl.bio.entity.factory.**XmlBioEntitiesFactory**- org.metaqtl.bio.entity.factory.**XmlGeneticMapFactory**- org.metaqtl.bio.entity.factory.**XmlOntologyFactory**
                - org.metaqtl.bio.util.**BioEntityNameComparator** (implements java.util.Comparator<T>)- org.metaqtl.bio.util.**BioLocusTypes**- org.metaqtl.bio.util.**BioUtilities**- org.metaqtl.graph.**ChromAxe**- org.metaqtl.**ChromCluster**- org.metaqtl.**ChromCluster.ClusterEdge**- org.metaqtl.**ChromCluster.ClusterGraph**- org.metaqtl.graph.**ChromGraph**- org.metaqtl.graph.**ChromLayer.CMrkTickPath**- org.metaqtl.graph.**ChromLayer.CMrkTickPoint**- org.metaqtl.**Chromosome**- org.metaqtl.adapter.**ChromosomeAdapter**- org.metaqtl.**CMarkerSequence**- org.metaqtl.main.**CmdLineParser**- org.metaqtl.main.**CmdLineParser.Option**
                                              - org.metaqtl.main.**CmdLineParser.Option.BooleanOption**- org.metaqtl.main.**CmdLineParser.Option.DoubleOption**- org.metaqtl.main.**CmdLineParser.Option.IntegerOption**- org.metaqtl.main.**CmdLineParser.Option.LongOption**- org.metaqtl.main.**CmdLineParser.Option.StringOption**
                                              - org.metaqtl.algo.**CMSAlgorithm**- org.metaqtl.factory.**DubiousLocusFactory**- org.metaqtl.algo.**EMAlgorithm**- org.metaqtl.**EMCriteria** (implements java.io.Serializable)- org.metaqtl.**EMResult**- org.metaqtl.factory.**EMResultFactory**- org.metaqtl.util.**GeneticMapProperties**- org.metaqtl.graph.**GraphFactory**- org.metaqtl.graph.**GraphShape**
                                                                - org.metaqtl.graph.**ChromShape**- org.metaqtl.graph.**QtlShape**
                                                                - org.metaqtl.algo.**HClustAlgorithm**- org.metaqtl.bio.util.**LGroupPosition** (implements org.metaqtl.bio.util.ILGroupPosition)
                                                                    - org.metaqtl.bio.util.**EstimatedLocusPosition**- org.metaqtl.bio.util.**StaticLocusPosition**
                                                                    - org.metaqtl.bio.util.**LocusPositionComparator** (implements java.util.Comparator<T>)- org.metaqtl.util.**LocusProperties**- org.metaqtl.**MapCluster**- org.metaqtl.factory.**MapDBFactory**- org.metaqtl.util.**MappingFunction**- org.metaqtl.algo.**MetaAlgorithm** (implements org.metaqtl.IMetaAlgorithm)
                                                                                - org.metaqtl.algo.**ClustAlgorithm**
                                                                                  - org.metaqtl.algo.**QtlClustAlgorithm**- org.metaqtl.algo.**QtlTreeAlgorithm**
                                                                                  - org.metaqtl.algo.**MetaMapAlgorithm**- org.metaqtl.algo.**QtlProjAlgorithm**
                                                                                - org.metaqtl.**MetaChrom**- org.metaqtl.**MetaDico**- org.metaqtl.factory.**MetaDicoFactory**- org.metaqtl.graph.**MetaGraph**- org.metaqtl.graph.**MetaGraphPar**- org.metaqtl.factory.**MetaGraphParFactory**- org.metaqtl.main.**MetaMain**
                                                                                              - org.metaqtl.main.**A2Xml**- org.metaqtl.main.**ConsMap**- org.metaqtl.main.**InfoMap**- org.metaqtl.main.**MapView**- org.metaqtl.main.**MetaDB**- org.metaqtl.main.**MMapView**- org.metaqtl.main.**MQTLView**- org.metaqtl.main.**QTLClust**- org.metaqtl.main.**QTLClustInfo**- org.metaqtl.main.**QTLModel**- org.metaqtl.main.**QTLProj**- org.metaqtl.main.**QTLTree**- org.metaqtl.main.**Xml2A**
                                                                                              - org.metaqtl.**MetaMap**- org.metaqtl.adapter.**MetaMapAdapter**- org.metaqtl.factory.**MetaMapSummaryFactory**- org.metaqtl.**MetaQtlAnalysis**- org.metaqtl.adapter.**MetaQtlAnalysisAdapter**- org.metaqtl.factory.**MetaQtlAnalysisFactory**- org.metaqtl.factory.**MetaQtlAnalysisModelFactory**- org.metaqtl.factory.**MetaQtlAnalysisSummaryFactory**- org.metaqtl.**MetaQtlData**- org.metaqtl.**MetaQtlModel**- org.metaqtl.factory.**MetaQtlModelFactory**- org.metaqtl.**MetaQtlResult**- org.metaqtl.**MultiFitResult**- org.metaqtl.bio.util.**NumberFormat**- org.metaqtl.numrec.**NumericalUtilities**- org.metaqtl.util.**OffspringManager**- org.metaqtl.util.**OffspringManager.HaldaneWaddingtonProportion**- org.metaqtl.graph.**PaletteColor**- org.metaqtl.bio.entity.bean.**PropertyBean**- org.metaqtl.**Qtl**- org.metaqtl.**Qtl.QTLCross** (implements java.io.Serializable)- org.metaqtl.**Qtl.QTLProj** (implements java.io.Serializable)- org.metaqtl.**Qtl.QTLStats** (implements java.io.Serializable)- org.metaqtl.**Qtl.QTLSupport** (implements java.io.Serializable)- org.metaqtl.**Qtl.QTLTrait** (implements java.io.Serializable)- org.metaqtl.adapter.**QtlAdapter**- org.metaqtl.graph.**QtlLayerBuffer**- org.metaqtl.**QtlPartition**- org.metaqtl.adapter.**QtlPartitionAdapter**- org.metaqtl.graph.**QtlUnit**- org.metaqtl.algo.**SVDAlgorithm**- org.metaqtl.bio.util.**TabulatedReader**- org.metaqtl.bio.util.**TabulatedWriter**- java.lang.Throwable (implements java.io.Serializable)
                                                                                                                                                                  - java.lang.Exception
                                                                                                                                                                    - org.metaqtl.main.**CmdLineParser.OptionException**
                                                                                                                                                                      - org.metaqtl.main.**CmdLineParser.IllegalOptionValueException**- org.metaqtl.main.**CmdLineParser.UnknownOptionException**
                                                                                                                                                                          - org.metaqtl.main.**CmdLineParser.NotFlagException**- org.metaqtl.main.**CmdLineParser.UnknownSuboptionException**
                                                                                                                                                                  - org.metaqtl.**Tree**- org.metaqtl.factory.**TreeFactory**- org.metaqtl.**TreeNode** (implements java.io.Serializable)

## Interface Hierarchy

- org.metaqtl.bio.**IBioAdaptable**
  - org.metaqtl.bio.**IBioAllele**- org.metaqtl.bio.**IBioCross**- org.metaqtl.bio.**IBioEntity**
        - org.metaqtl.bio.**IBioAllele**- org.metaqtl.bio.**IBioCross**- org.metaqtl.bio.**IBioGenome**- org.metaqtl.bio.**IBioIndividual**- org.metaqtl.bio.**IBioLGroup**- org.metaqtl.bio.**IBioLocus**- org.metaqtl.bio.**IBioOntology**- org.metaqtl.bio.**IBioOntologyTerm**- org.metaqtl.bio.**IBioPopulation**
                          - org.metaqtl.bio.**IBioCross**
        - org.metaqtl.bio.**IBioGenome**- org.metaqtl.bio.**IBioIndividual**- org.metaqtl.bio.**IBioLGroup**- org.metaqtl.bio.**IBioLocus**- org.metaqtl.bio.**IBioOntology**- org.metaqtl.bio.**IBioOntologyTerm**- org.metaqtl.bio.**IBioPopulation**
                      - org.metaqtl.bio.**IBioCross**
  - org.metaqtl.bio.**IBioAdapter**- org.metaqtl.bio.entity.**IBioConstants**- org.metaqtl.bio.**IBioCrossTypes**- org.metaqtl.bio.**IBioEntityTypes**- org.metaqtl.**IBioGenomeProperties**- org.metaqtl.bio.**IBioIndividualTree**- org.metaqtl.**IBioLGroupProperties**- org.metaqtl.**IBioLocusProperties**- org.metaqtl.bio.entity.**IGeneticMapProperties**- org.metaqtl.bio.util.**ILGroupPosition**- org.metaqtl.**IMetaAlgorithm**- org.metaqtl.**IMetaQtlConstants**- org.metaqtl.bio.**IOntologyTermProperties**- org.metaqtl.numrec.**NumericalUtilities.Function**- org.metaqtl.numrec.**NumericalUtilities.NRFunction**

---


|  |  |  |
| --- | --- | --- |
| **Overview** \| Package \| Class \| Use \| **Tree** \| **Deprecated** \| **Index** \| **Help** | |  |
| PREV   NEXT | **FRAMES**    **NO FRAMES**     **All Classes** |  |


---
